# High-sensitivity dynamic diffuse fluorescence tomography system for fluorescence pharmacokinetics

**DOI:** 10.1117/1.JBO.27.4.046002

**Published:** 2022-04-22

**Authors:** Limin Zhang, Nan Cheng, Han Liu, Yingxue Pan, Yanqi Zhang, Feng Gao

**Affiliations:** a Tianjin University, College of Precision Instrument and Optoelectronics Engineering, Tianjin, China; b Tianjin Key Laboratory of Biomedical Detecting Techniques and Instrument, Tianjin, China; c Tianjin University, Tianjin International Engineering Institute, Tianjin, China; d Tianjin Medical University, School of Medical Imaging, Tianjin, China

**Keywords:** diffuse fluorescence tomography, dynamic measurement, indocyanine green, lock-in photon counting

## Abstract

**Significance:**

Dynamic diffuse fluorescence tomography (DFT) can recover the static distribution of fluorophores and track dynamic temporal events related to physiological and disease progression. Dynamic imaging indocyanine green (ICG) approved by the food and drug administration is still under-exploited because of its characteristics of low quantum yield and relatively rapid tissue metabolism.

**Aim:**

In order to acquire the ICG tomographic image sequences for pharmacokinetic analysis, a dynamic DFT system was proposed.

**Approach:**

A fiber-based dynamic DFT system adopts square-wave modulation lock-in photon-counting scheme and series-parallel measurement mode, which possesses high sensitivity, large dynamic range, high anti-ambient light ability in common knowledge, as well as good cost performance. In order to investigate the effectiveness of the proposed system, the measurement stability and the anti-crosstalk—a crucial factor affecting the system parallelization—were assessed firstly, then a series of static phantoms, dynamic phantoms and *in vivo* mice experiments were conducted to verify the imaging capability.

**Results:**

The system has the limited dynamic range of 100 dB, the fluctuation of photon counting within 3%, and channel-to-channel crosstalk ratio better than 1.35. Under the condition of a sufficient signal-to-noise ratio, a complete measurement time for one frame image was 10.08 s. The experimental results of static phantoms with a single target and three targets showed that this system can accurately obtain the positions, sizes, and shapes of the targets and the reconstructed images exhibited a high quantitativeness. Further, the self-designed dynamic phantom experiments demonstrated the capability of the system to capture fast changing fluorescence signals. Finally, the *in vivo* experiments validated the practical capability of the system to effectively track the ICG metabolism in living mice.

**Conclusions:**

These results demonstrate that our proposed system can be utilized for assessing ICG pharmacokinetics, which may provide a valuable tool for tumor detection, drug assessment, and liver function evaluation.

## 1 Introduction

Diffuse fluorescence tomography (DFT) with the merits of non-invasiveness, radiation-free, high sensitivity, and low cost has become a promising *in vivo* optical imaging technique for disease diagnosis, treatment monitoring, and drug screening.^1^^–^^3^ Compared with most of the commercially available fluorescence imaging devices that offer the planar hierarchy distribution of fluorescence, DFT can be used for localization and quantification of the three-dimensional distribution of near-infrared (NIR) fluorophore.^4^^,^^5^ Currently, an under-exploited benefit is its ability to acquire data at high speed for real-time monitoring the fast-changing fluorescence signals. This enables DFT to both recover static distributions of fluorophore and track dynamic temporal events related to physiological and disease progression.^6^^,^^7^

NIR contrast agents are suitable for optical imaging applications with high signal-to-background ratio and deep tissue penetration due to low absorption in the NIR spectrum. Among the various commercially available optical imaging agents, indocyanine green (ICG) with the absorption and emission peaks at ∼780 and at 830 nm, respectively is the workhorse agent for NIR imaging, because it is approved by the United States Food and Drug Administration for use in humans.^8^^–^^10^ If injected intravenously, ICG diffuses into tumor tissue by binding with plasma proteins due to its enhanced permeability and retention effect. In addition, ICG as an exogenous organic anion is eliminated exclusively by the liver, which is expected to demonstrate different clearance rate between healthy and impaired livers.^11^^,^^12^ Therefore, developing dynamic DFT technique to obtain a sequence of time-varying ICG concentration images and further combining with pharmacokinetic analysis methods to extract pharmacokinetic parameters may provide enhanced contrast and specificity due to differences in pharmacokinetic properties between diseased and healthy tissues.

Generally, DFT systems can probe light by optical fibers combined with highly sensitive photoelectric sensors [photomultiplier tubes (PMT) or avalanche photodiodes], or alternatively by sensitive charge-coupled device (CCD) cameras.^13^^–^^20^ Compared with CCD camera detection mode, fiber-based detection combining with high-sensitive photoelectric sensors and photon-counting technique allows higher detection sensitivity and larger dynamic measurement range, which is significantly beneficial to probing ICG suffering low quantum yield, especially after a period of metabolism in tissue. At present, the pharmacokinetics of ICG has been studied to assess tumor issues and mouse liver function with the support of fiber-based DFT systems. Intes et al.^15^ studied the uptake of ICG by breast tumors, and the experimental results showed that the malignant cases exhibited slower uptake and outflow compared with healthy tissue, and differing pathologies demonstrated different dynamical features. Similarly, Alacam et al.^16^ reconstructed the ICG pharmacokinetic-rate images of breast tumors, which showed the ICG pharmacokinetic rates ($k_{\text{in}}$ and $k_{\text{out}}$ govern the leakage into and the drainage out of the extracellular–extravascular space) from the tumor region and outside the tumor region were statistically different. Zhang et al.^17^ obtained the pharmacokinetic parameters (uptake and excretion rates) of ICG in healthy mouse liver. Our group built a fiber-based DFT system with computed tomography (CT)-analogous scanning mode to study the metabolisms of ICG in healthy mouse liver as well as ICG-loaded nanoparticles in mouse tumor tissue.^18^^–^^20^

Fiber-based DFT systems were commonly configured with 16 source and detection positions distributed around a circular holder at equal separations. When one source was illuminated, 16 detection data were collected on boundary simultaneously or sequentially by optical switching according to the detector number. In our previously reported system, a fiber-based DFT system with analogous to CT scan mode was proposed.^18^ Briefly, a collimated light source with 780 nm wavelength was employed to excite ICG, and four parallel PMT photon-counting-channels were optically switched to eight detection sites. By rotating the object from 0 deg to 360 deg with an interval of 22.5 deg analogous to CT scan, the acquisition time for collecting one complete frame of the projection data (16×8) was ∼1.1  min under the consideration of the rotating object (2 s), photon counting (1 s), and optical switching time (0.02 s), which is equal to the measurement time of the system proposed by Liu et al.,^13^ and is longer than that proposed by Alacam et al.^16^ (8.8 s) without optical switch and with shorter photon-counting time (0.5 s).^19^

In this work, to further improve the capability of dynamic measurement under the consideration of cost and efficiency, a unique designing scheme was proposed by adopting digital lock-in photon-counting technique for realizing simultaneous irradiation of multiple light sources and parallel detection of multiple detectors. To validate the effectiveness of the proposed system, a series of static and dynamic phantom experiments, as well as *in vivo* experiments were conducted.

## 2 Materials and Methods

### 2.1 Dynamic DFT System

**Fig. 1:**
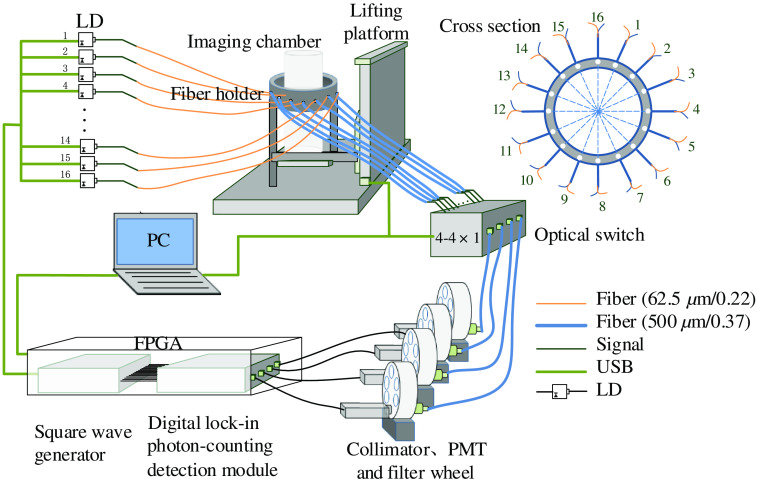
Schematic of dynamic DFT system.

As illustrated in Fig. 1, the proposed dynamic DFT system consists of a light source unit for generating continuous-wave laser light, a detection unit for detecting the excitation and emission lights, and a user interface unit for controlling and visualizing imaging results.

In this system, 16 pig-tailed laser diodes (L785P25, Thorlabs) at 785 nm wavelength driven by a constant current source module are controlled by square wave generator implemented on a field-programmable gate-array (FPGA, DE0-Nano-Soc Kit with Cyclone V, Terasic) hardware with the frequency modulation ranges from 0 to 10 kHz. Sixteen source fibers having a core diameter of 62.5  μm and a numerical aperture (NA) of 0.275 coupled with 16 detection fibers having a core diameter of 500  μm and a NA of 0.370 are mounted into the fiber holders with a uniform distribution around a cylindrical imaging chamber fixed on a motorized translation stage. The source fibers conduct the lights to illuminate the object fixed in the imaging chamber. Simultaneously the transmitted lights on the object boundary are collected by detector fibers and directed into an integrated module of programmable 4-1×4 fiber optic switch (FSW4-1×4-MM-C, Guilin Institute of Optical Communications, China). The light outputs from the fiber optic switch are collimated using collimators (FC230FC-B,Thorlabs) for normal incidence to four six-hole motorized filter wheels (FF01-832/37-25, Semrock) where one of the holes houses a single-band pass filter (FEL0700, Thorlabs) with the transmittance >95% at 830±5  nm and <0.01% at 780±5  nm for blocking excitation light. In principle, the fluorescence emission and excitation signals are detected with and without the filter stack in place, respectively. Subsequently, the light signals are fed into four PMT counting heads (H8259-02e, Hamamatsu, Japan) having a count linearity of $2.0\times 10^{6}\;s^{-1}$ and a dark count of $400\;s^{-1}$, and thereby a limited dynamic range of 100 dB, and are converted into electrical pulses corresponding to the PMT single-electron responses. Simultaneously, the collected composite signals are demodulated by digital lock-in photon-counting detection module. The whole setup is controlled by a LabVIEW-programmed graphical user interface.

In this dynamic DFT system, lock-in photon-counting technique were employed to obtain high sensitivity, large dynamic range, and high ability to reject ambient light as a common knowledge.^21^ In addition, serial-parallel detection mode was proposed to obtain a balance between measurement speed and cost-effectiveness. In the following, the detailed design principle was introduced.

#### 2.1.1 Serial-parallel measurement mode

**Fig. 2:**
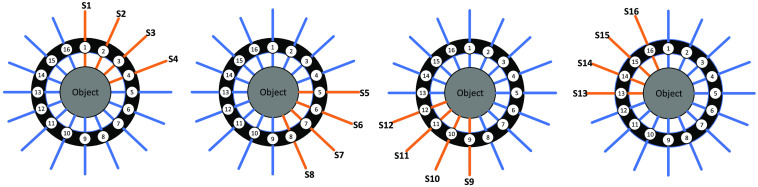
Schematic of the light excitation and detection processes.

The light excitation and detection processes adopting serial-parallel measurement mode are shown schematically in Fig. 2. The detailed process for a complete measurement can be described as follows. First, four modulated light sources (nos. 1 to 4) illuminate the object simultaneously, and the other positions divided into three groups (nos. 5 to 8, nos. 9 to 12, and nos. 13 to 16) detect the transmission light in turn, by a 4-1×4 optical switch. In the same manner, nos. 5 to 8, nos. 9 to 12, and nos. 13 to 16 light sources are modulated, respectively, and the other three groups are taken as detection positions accordingly. Consequently, a complete measurement can acquire a total number of 16×12 detection data for one frame image reconstruction, and the measurement time can be calculated with the following equation

$$T=3(T_{i}+T_{s})\times 4,$$ (1)

where $T_{i}$ is the integration time of digital lock-in photon-counting module; $T_{s}$ is the switching time of optical switch; the constants 3 and 4 denote the switch times of detection and source fibers, respectively.

#### 2.1.2 Square-wave modulation lock-in photon-counting scheme

For enabling simultaneous multichannel excitation and detection, phase-lock-in technique with square wave modulation was employed and primarily implemented by the digital-phase-detection (DPD) blocks in the system. For each measurement, four laser diodes in a group are switched “ON” and “OFF” by the four square-wave modulation signals with a duty cycle of 50% at the frequencies of 3.2, 3.6, 4.0, and 4.4 kHz, respectively. Meanwhile, they are sent to the lock-in photon-counting module as the reference signals of “1” weights for demodulation. Compared with sine-wave modulation scheme with the nonlinearity caused by the error of phase modulation, the square-wave modulation would not introduce nonlinearity error ascribe to only digital operations involved in the demodulation process, and more importantly, would greatly simplified the DPD block hardware.

**Fig. 3:**
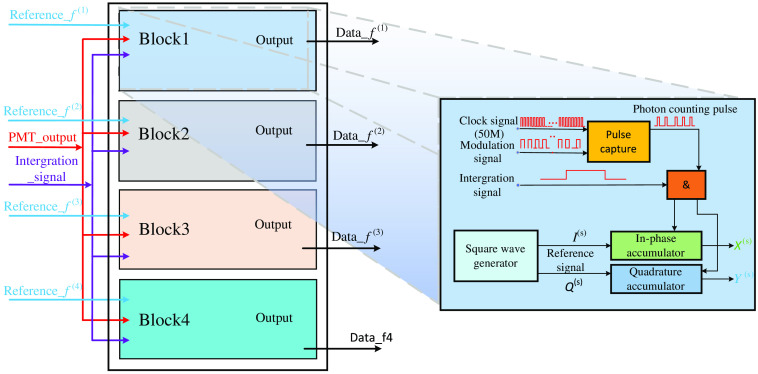
Block diagram of digital-phase-detection based on reference-weighted counting strategy.

As illustrated in Fig. 3, each PMT-connected demodulator (with regards to a detection position) in the lock-in photon-counting module is configured with four DPD blocks to maximally discriminate the component signals at four modulation frequencies respectively, from the received composite light signal. The intensity of the demodulated component signal is achieved by the multi-periodic reference-weighted counting (RWC) strategy in the DPD block, as shown on the right of Fig. 3, where the reference weights at the time of the PMT outputs are accumulated for multiple periods. To eliminate the initial time-shift uncertainty of the signal, a pair of in-phase $I^{\left(s\right)}(t)$ and quadrature $Q^{\left(s\right)}(t)$ reference signals having the same frequency $f^{\left(s\right)}$ (s=1, 2, 3, and 4) are introduced into each digital DPD block. These two reference signals are mathematically regarded as bipolar square-wave of “1” weights in the RWC operations, and can be expressed in terms of the Fourier series, which are given by

$$\{\begin{array}{l} I^{\left(s\right)}(t)=\frac{4}{\pi }\overset{\infty }{\underset{n=1}{\sum }}\frac{{\left(-1\right)}^{n-1}}{2n-1}\;\cos[(2n-1)2\pi f^{\left(s\right)}t]\\ Q^{\left(s\right)}(t)=\frac{4}{\pi }\overset{\infty }{\underset{n=1}{\sum }}\frac{{\left(-1\right)}^{n-1}}{2n-1}\;\sin[(2n-1)2\pi f^{\left(s\right)}t] \end{array}.$$ (2)

**Fig. 4:**
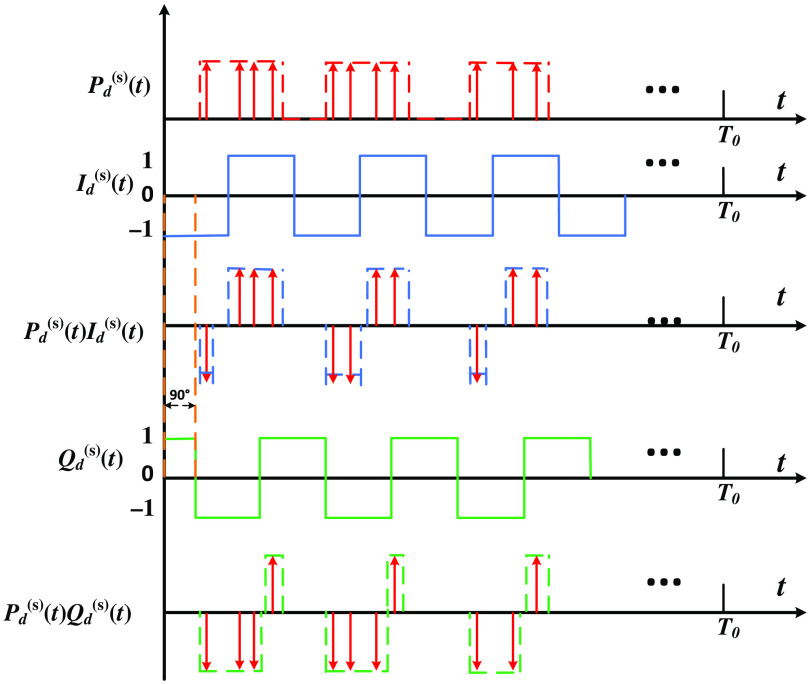
Diagram of the multi-periodic RWC operation with an integration time of $T_{0}$.

The RWC in the digital DPD is mathematically equivalent to the multiplication–integration operations in the analog DPD, where the operations can be conducted by adding or subtracting “1” (multiplication) for multiple periods (integration) depending on the reference phase of the PMT pulse occurrences $P_{d}^{\left(s\right)}(t)$, as illustrated in Fig. 4, where the up and down arrows indicate addition and subtraction of “1.” $X_{d}^{\left(s\right)}$ (in-phase) and $Y_{d}^{\left(s\right)}$ (quadrature) regard to the detection position d (1, 2, …, 12), is expressed as

$$X_{d}^{\left(s\right)}=\int_{0}^{T_{0}}P_{d}^{\left(s\right)}(t)I_{d}^{\left(s\right)}(t)dt=\{\begin{array}{cc} A_{d}^{\left(s\right)}(\frac{1}{2}-2\tau_{d}^{\left(s\right)}f^{\left(s\right)}), & 0<\tau_{d}^{\left(s\right)}\leq \frac{1}{2f^{\left(s\right)}}\\ A_{d}^{\left(s\right)}(2\tau_{d}^{\left(s\right)}f^{\left(s\right)}-\frac{3}{2}), & \frac{1}{2f^{\left(s\right)}}<\theta \leq \frac{1}{f^{\left(s\right)}} \end{array},$$ (3)

$$Y_{d}^{\left(s\right)}=\int_{0}^{T_{0}}P_{d}^{\left(s\right)}(t)Q_{d}^{\left(s\right)}(t)dt=\{\begin{array}{ll} A_{d}^{\left(s\right)}(2\tau_{d}^{\left(s\right)}f^{\left(s\right)}), & 0<\tau_{d}^{\left(s\right)}\leq \frac{1}{4f^{\left(s\right)}}\\ A_{d}^{\left(s\right)}(2\tau_{d}^{\left(s\right)}f^{\left(s\right)}-1), & \frac{1}{4f^{\left(s\right)}}<\theta \leq \frac{3}{4f^{\left(s\right)}}\\ A_{d}^{\left(s\right)}(2-2\tau_{d}^{\left(s\right)}f^{\left(s\right)}), & \frac{3}{4f^{\left(s\right)}}<\tau_{d}^{\left(s\right)}\leq \frac{1}{f^{\left(s\right)}} \end{array},$$ (4)

where $A_{d}^{\left(s\right)}$ and $\tau_{d}^{\left(s\right)}$ represent the intensity and the initial time-shift, respectively.

Finally, the photon-count $M_{d}^{\left(s\right)}$ can be calculated from the obtained $X_{d}^{\left(s\right)}$ and $Y_{d}^{\left(s\right)}$ as the measured component signal intensity and the related equation is

$$M_{d}^{\left(s\right)}=|X_{d}^{\left(s\right)}|+|Y_{d}^{\left(s\right)}|\propto A_{d}^{\left(s\right)}.$$ (5)

Before conducting imaging experiments, we have assessed the system performances in terms of stability and anti-crosstalk-a crucial factor affecting the system parallelization. The results showed that the photon counts fluctuated within 3% during 4 h period, and the channel-to-channel crosstalk ratio was better than 1.35%, suggesting the system demonstrated good performance. The detailed evaluation methods can be referenced in our previous work.^22^ For the following experiments, the integration time of lock-in photon-counting module was determined to be 0.8 s to obtain a sufficient signal-to-noise ratio, and the switching time of optical switch was set to 0.04 s. Thus, a complete measurement time for one frame image was 10.08 s, according to Eq. (1).

### 2.2 DFT Reconstruction Method

NIR light transport in biological tissues can be described by the diffusion equation. For fluorescence tomographic imaging with continuous wave light source, the photon transport model can be described by a coupled diffusion equation, which is given by^23^

$$\left\{ \begin{array}{l} \left[\nabla \cdot D_{x}(r)\nabla -\mu_{ax}(r)c]\Phi_{x}(r,r_{s})=-\delta (r-r_{s}\right)\\ \left[\nabla \cdot D_{m}(r)\nabla -\mu_{am}(r)c]\Phi_{m}(r,r_{s})=-c\Phi_{x}(r,r_{s})\eta \mu_{af}(r\right) \end{array},\right.$$ (6)

where subscripts x and m denote the excitation and emission wavelengths, respectively; $\Phi_{v}(r,r_{s})$ (v∈[x,m]) is the photon density; the optical parameters involved are the absorption coefficient $\mu_{av}(r)$, the reduced scattering coefficient $\mu_{sv}^{\prime }(r)$ and the diffusion coefficient $D_{v}(r)=c/3[\mu_{av}(r)+\mu_{sv}^{\prime }(r)]$; c is the speed of light in medium; the fluorescence parameter is the fluorescent yield $\eta \mu_{af}(r)$; and η is the quantum efficiency (0.016, ICG). These quantities are generally the functions of the position vector r.

For fluorescence tomographic reconstructions, normalized Born ratio $I_{nb}(r_{d},r_{s})$ that divides fluorescence measurements ${\overset{⌢}{I}}_{m}(r_{d},r_{s})$ with corresponding excitation measurements ${\overset{⌢}{I}}_{x}(r_{d},r_{s})$ is utilized. The related equation is

$$I_{nb}\left(r_{d},r_{s}\right)=\frac{{\overset{⌢}{I}}_{m}(r_{d},r_{s})}{{\overset{⌢}{I}}_{x}(r_{d},r_{s})}=\frac{\int d^{3}rcG(r_{d},r)\Phi_{x}(r,r_{s})\eta \mu_{af}(r)}{I_{x}(r_{d},r_{s})},$$ (7)

where $I_{x}(r_{d},r_{s})$ is the calculated excitation flux at $r_{d}$ for a source at $r_{s}$; $G(r_{d},r)$ is the Green’s function describing light propagation at the fluorescence wavelength.

Equation (7) can be degenerated into a matrix equation $I_{nb}=\mathrm{WX}$, where W is the weighted matrix of size $N_{d\times s}\times N_{\text{voxels}}$, where $N_{d\times s}$ is the number of measurements and $N_{\text{voxels}}$ is the number of nodes of the finite element method meshes; X is the fluorescence yield to be reconstructed. In this paper, the fluorescence yield images were recovered relayed on algebraic reconstruction technique, where the iteration number and relaxation parameter were empirically determined to be 30 and 0.2, respectively.^23^

### 2.3 Pharmacokinetic Analysis Method

Based on dynamic DFT measurement and reconstruction algorithm, a sequence of fluorescence yield images can be acquired. Further, the ICG concentration C(t) (μM) at time t can be calculated from fluorescence yield ($\eta \mu_{af}(t)$) reconstruction by using the equation^24^

$$\mu_{af}(t)=\ln\;10\xi C(t),$$ (8)

where ξ is the extinction coefficient of ICG ($0.013\;{\mathrm{mm}}^{-1}\;\mu M^{-1}$).^25^

For the *in vivo* experiment involving assessing liver function, the ICG kinetics in the livers can be monitored by measuring the ICG concentration-time curves that can be described by a biexponential fitting that is expressed as^26^

C(t)=−A exp(−αt)+B exp(−βt), (9)

where A and B (unit: a.u.) are the zero-time intercepts, representing the initial hepatic ICG concentration; α and β (unit: $\min^{-1}$) are the uptake and excretion rates representing ICG influx to the liver and disappearance from the liver, respectively, which are used to quantitatively assess liver function.

### 2.4 Experiments Methods

#### 2.4.1 Static phantom experiments

**Fig. 5:**
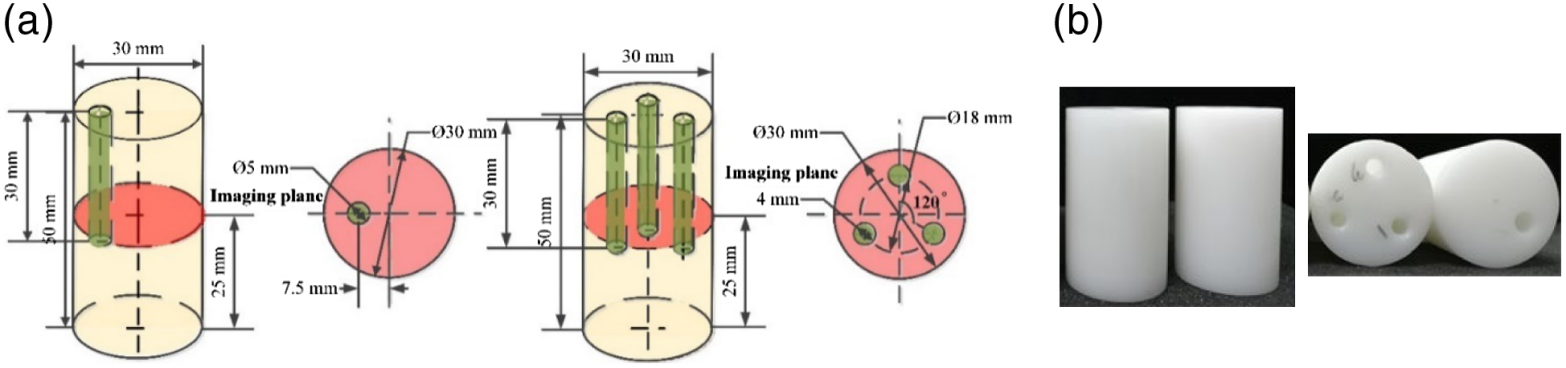
(a) Sketches of phantoms with a single target and three targets for static experiment. (b) Photos of the phantoms.

Figure 5 shows the sketches and photos of phantoms with a single target and three targets for static experiments. The phantoms were made of polyformaldehyde, which had the scattering characteristics similar to biological tissue. The absorption and reduced scattering properties were determined to be 0.0034 and $0.08\;{\mathrm{mm}}^{-1}$ at 785 nm, using time-resolved spectroscopy method. The diameter and height of the phantoms were 30 and 50 mm, respectively. To mimic a target tumor in biological tissue, a mixture of 1% Intralipid and ICG (Liaoning Tianyi Biological Pharmaceutical Co., Ltd., China) was placed in a circular hole drilled in the phantom. Here, two types of phantoms were designed: a single-target phantom with a hole of 5 mm diameter, 30 mm depth, and 7.5 mm off-center; a three-target phantom with three holes of 4 mm diameter and 35 mm depth, locating at the positions that were 9 mm away from the central axis of the phantom and 120 deg separating angles among them. In the single target experiment, the hole was filled with the solution of 1% Intralipid mixed with 0.5, 1.0, and 2.0  μmol/L ICG, respectively. Furthermore, in order to verify the capability of imaging multiple targets simultaneously, two types of three-target phantoms were configured. One phantom was filled with 1% Intralipid mixed with 3.0  μmol/L ICG in all three holes. The other was filled with 1% Intralipid mixed with 0.5 (no. 1 target), 1.0 (no. 2 target), and 2.0  μmol/L (no. 3 target) ICG in three holes, respectively.

#### 2.4.2 Dynamic phantom experiments

**Fig. 6:**
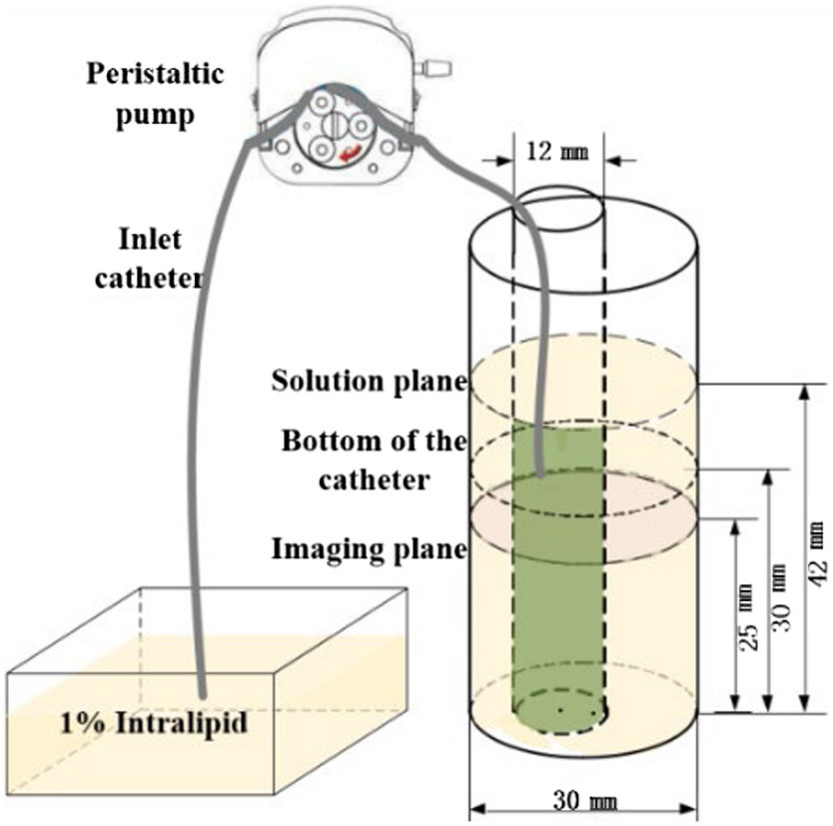
Schematic of the dynamic phantom.

Figure 6 shows a self-designed dynamic phantom. It was made of a cylindrical polyformaldehyde chamber of 30-mm diameter, filled with 1% Intralipid as the background and a cylindrical tube of 12-mm diameter filled with a mixture of 1% Intralipid solution and ICG as the target. To simulate the ICG concentration change process in the target region, 1% Intralipid fresh solution was pumped into the target tube, using a peristaltic pump-controlled silicone catheter (BT100-02, China). For evenly mixing the solution in the target cylinder during a limited time, the inlet catheter was placed at the height of 30 mm below the solution level (42 mm), and the imaging plane was selected at the height of 25 mm below but close to the inlet catheter.

According to the instruction of the peristaltic pump, the flow speed $V_{\text{pump}}$ (unit: mL/min) of the silicone catheter and the concentration of ICG can be calculated using the equation

$$C_{i}=C_{0}V_{0}/(V_{0}+i\alpha V_{\text{pump}}),$$ (10)

where $V_{\text{pump}}$ (rpm) denotes the rotation speed of the peristaltic pump and α is a constant determined by the silicone catheter type. $C_{0}$ and $V_{0}$ are the initial concentration and volume of ICG, respectively. Under the same $V_{\text{pump}}$, the change rate of ICG concentration can be altered by setting different initial ICG concentration.

For performing dynamic experiment, 1% Intralipid mixed with 1.0  μmol/L ICG was adopted as the initial target concentration. The rotation speed of peristaltic pump was adjusted to 4 rpm, and α was select to be 0.00105, which means the injection speed of the solution was 0.0252  ml/min. The whole detection process lasted ∼18  min for 104 complete measurements.

#### 2.4.3 *In vivo* experiments

**Fig. 7:**
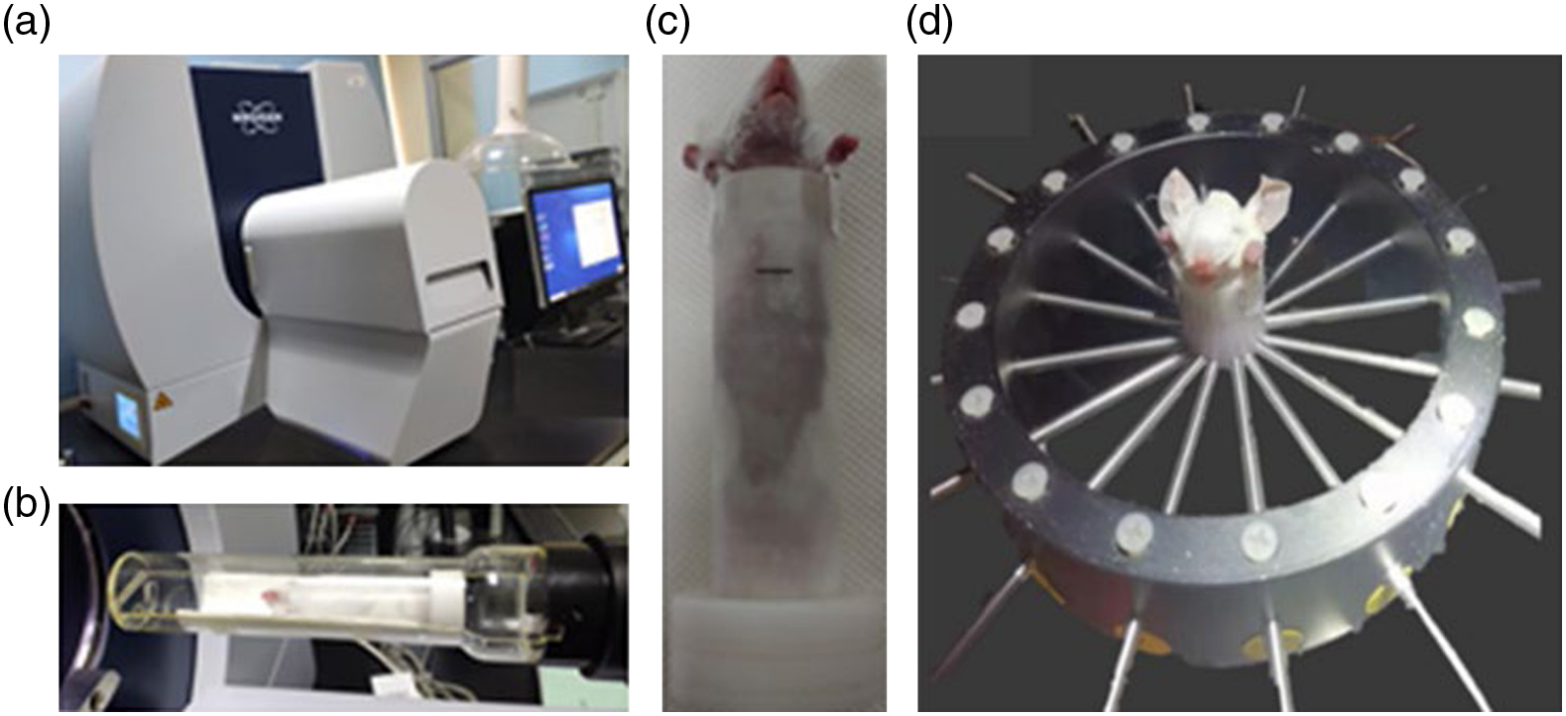
Pictures of *in vivo* experiments: (a) photo of micro-CT system, (b) the *in vivo* mouse in imaging chamber for micro-CT measurement, (c) the mark on chamber surface for image co-registration of DFT and micro-CT, and (d) DFT measurement.

Figure 7 illustrates the pictures of *in vivo* experiments-based micro-CT system and dynamic DFT system. In the *in vivo* experiments, three four-week-old Kunming mice (weighing about 13 to 16 g) were employed. All the protocols for animal experiments were carried out according to the guidelines of the Council for the Purpose of Control and Supervision of Experiments on Animals Ministry of Health, Government of China. The experimental process can be briefly described as follows. Before performing the dynamic measurement, the mouse was anesthetized with 4% chloral hydrate at a dose of 0.01  mL/g body weight through the abdominal cavity and removed hair by using depilatory cream for better detection of light signal. Then a bolus of ICG (0.6 to 0.7 mL, 50  μg/mL) was injected via tail vein. Subsequently, the mouse was fixed in the imaging chamber and filled the interspace with 1% Intralipid. According to anatomical principle, we ascertained the imaging plane of mouse liver at 0.5 to 1 cm blow the xiphoid and slightly marked the corresponding chamber surface. It is worth mentioning that the uptake rate of ICG in mice is rapid enough, which usually takes about 3 min to accumulate to the peak of ICG content in liver after injection. Thus, the dynamic probe started 3 min later after the ICG injection. In order to capture the metabolic process of ICG in liver, 120 complete measurements were implemented continuously, ∼20  min. To verify the reliability of liver position obtained by DFT reconstruction, a micro-CT imaging system (SkyScan 1276, bruker, German) was used to obtain the anatomical structure. Before micro-CT imaging, an iron anchor point was plastered on the previous mark position of the chamber surface to guarantee the same imaging plane for DFT and micro-CT. The micro-CT imaging began 30 min after intravenous administration of a contrast agent at a dose of 0.4  mL/mouse, produced at Tianjin Medical University. It is worth mentioning that the bottom of the imaging chamber is detachable. Thus, the tail vein injections of ICG and liver contrast agent were able to be performed in the same chamber. For *in vivo* imaging, the $\mu_{av}(r)$ and $\mu_{sv}^{\prime }(r)$ are set to 0.03 and $1.0\;{\mathrm{mm}}^{-1}$, respectively, corresponding with the average values of bulk tissue.^27^

## 3 Results and Discussions

### 3.1 Static Phantom Experimental Results

**Fig. 8:**
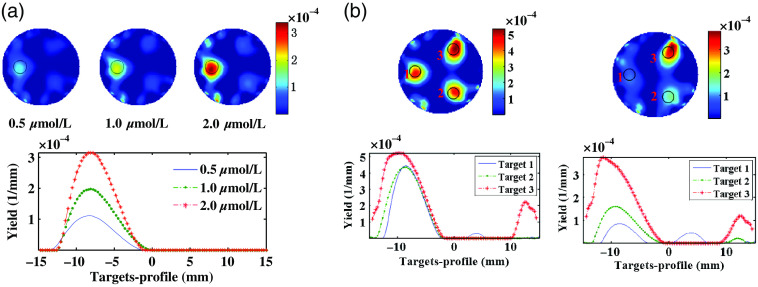
(a) Reconstructed fluorescence yield images of single-target phantoms with 0.5, 1.0, and 2.00  μmol/L ICG, respectively, and the corresponding target profiles. (b) Reconstructed fluorescence yield images of three-target phantoms and the corresponding target profiles: all the three targets with 4  μmol/L ICG(left) and the three targets with 0.5, 1.0, and 2.0  μmol/L ICG, respectively.

Figure 8(a) illustrates the reconstructed yield images in imaging plane and the corresponding x-profiles at y=0  mm. We can observe that the reconstructed target was clearly distinguishable from the background and displayed a good agreement with the true target (real line) in terms of the localization and size. We can also see that the reconstructed target region became more remarkable with the ICG concentration increase, and the x-profile plotted along the horizontal line passing through the centers of the target quantitively depicted that the fluorescence yield increased in proportion to ICG concentration. Figure 8(b) shows the reconstructed yield images, and the profiles along the lines passing through the centers of the phantoms and the targets. From the reconstructed fluorescence yield images of the three-target phantoms and the corresponding target profiles, we can observe that the proposed system can still disclose the locations and sizes of the three targets for both types of phantoms. For the phantom with three targets having the same ICG concentration, the reconstructed fluorescence yields of three targets demonstrated a good consistency in quantitativeness, except that the target 3 is slightly larger. For the three targets with different ICG concentration, it is evident that the reconstructed fluorescence yield increases with the increase of ICG concentration. In addition, we find that the fluorescence yield ratio calculated using the maximum of the target-profile approximately conforms to 1:2:4, which is consistent with the theoretical value. These results demonstrate that the system possesses high sensitivity and quantitativeness.

### 3.2 Dynamic Phantom Experimental Results

**Fig. 9:**
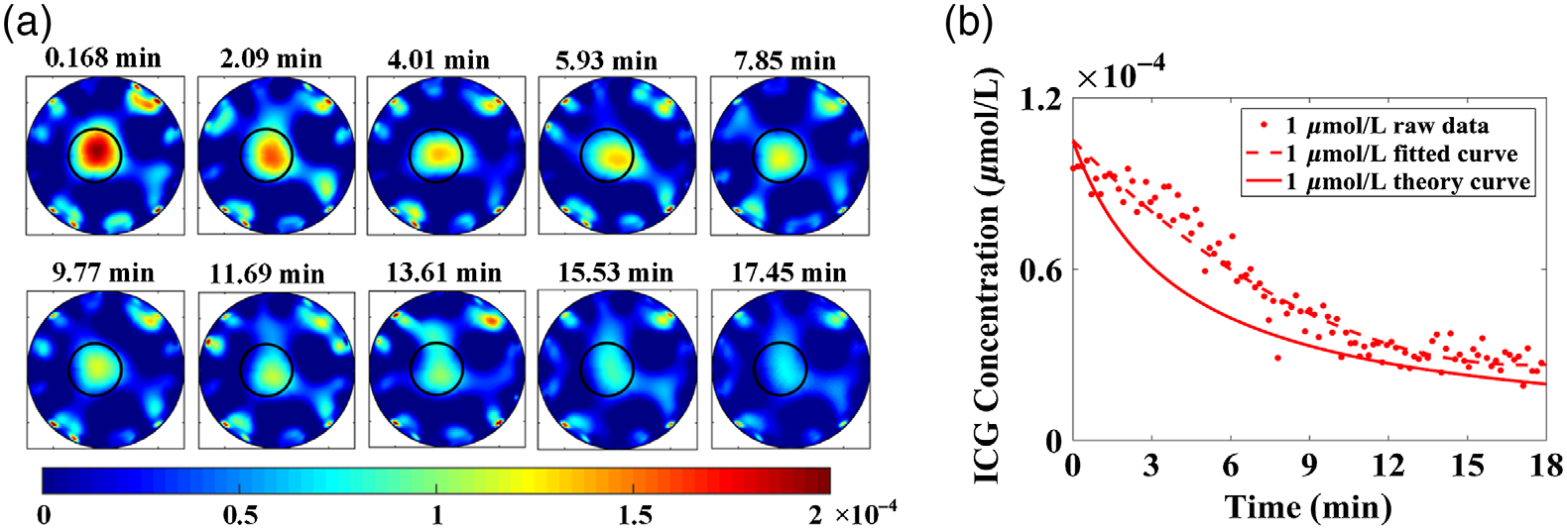
(a) The fluorescence yield images reconstructed from 0.168 min to 17.45 min with a time interval of 1.92 mins at the initial ICG concentrations of 1  μmol/L; (b) The raw data, fitted curve and theory of ICG concentration versus time.

Figure 9(a) typically displays the fluorescence yield images reconstructed from 0.168 to 17.45 min with a time interval of 1.92 min at the initial ICG concentration of 1  μmol/L. We can see that the reconstructed target is consistent with the true location and size of the target (black circle). Figure 9(b) shows the theory curve (real line) of the ICG concentrations, the raw data (markers) calculated by the average ICG concentrations in the target region and the fitted curve (dotted line) versus the measurement time. It can be seen that the reconstructed ICG concentration demonstrated a declining trend with time, and the reconstructed values became closer to theoretical values with the measurement time increased, indicating the capacity of the dynamic DFT system to capture the rapidly changing fluorescence signals. However, the reconstructed values partly deviated from the theory values, which is mainly because that the exchange of the solution in the target needed some time. The fluorescence yield values in the dynamic phantom experiments could not decrease as fast as the theoretical calculating values, especially for the situation that the ICG concentration is higher.

### 3.3 *In Vivo* Experimental Results

**Fig. 10:**
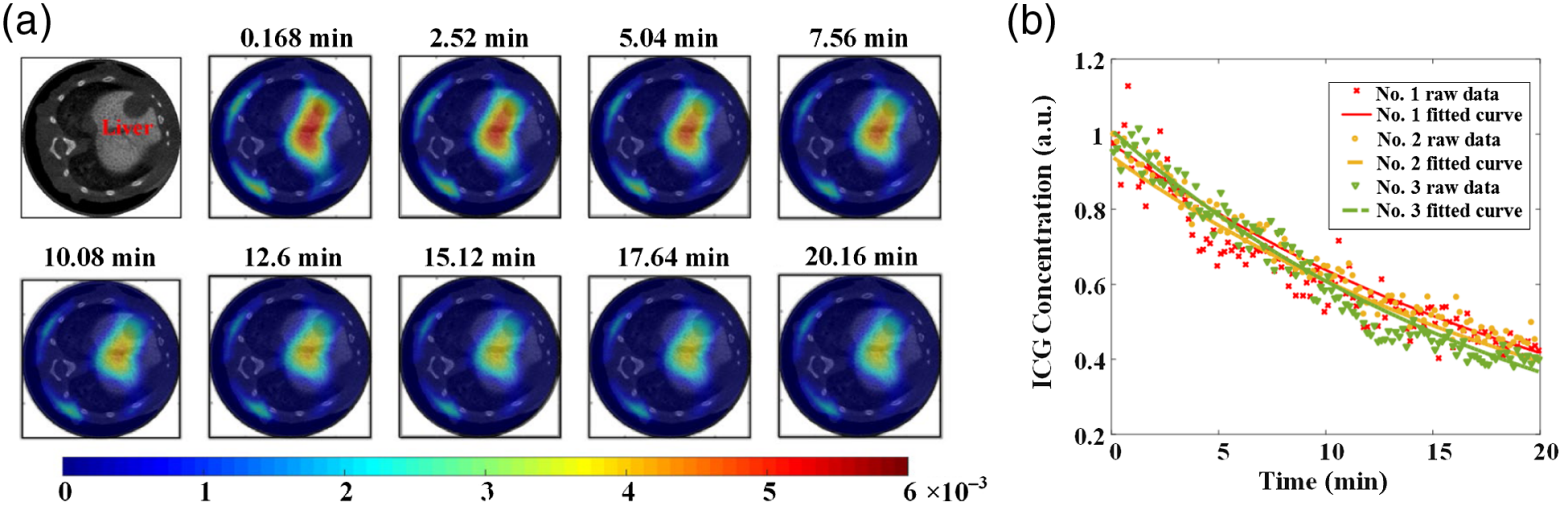
(a) A slice of the micro-CT images corresponding to the DFT measurement plane and the overlays of the anatomical images and the corresponding fluorescence tomographic images sequences with an interval of 14 image frames. (b) The plots of time-course changes in the normalized ICG concentrations and the corresponding fitted curves.

As mentioned previously, the dynamic measurement started after 3 min post-injection. Thus, only the pharmacokinetic parameters of B and β were extracted based on the second exponential part of Eq. (9). Figure 10(a) shows a slice of the micro-CT images corresponding to the DFT measurement plane and typically illustrates the overlays of the anatomical images and the corresponding fluorescence tomographic image sequences with an interval of 14 image frames. For reflecting the average metabolic process of ICG in livers, the mean values of the ICG concentrations in liver were calculated. Figure 10(b) shows the plots of time-course changes in the normalized ICG concentrations and the corresponding fitted curves that can be obtained by an exponential-curve-fitting method.

As can been seen from Fig. 10(b), the ICG concentrations decrease with the ICG metabolism in liver and the fitted curves are close to each other. To quantitatively assess the pharmacokinetic parameters of each mouse, the fitted parameters (B and β) for each mouse are listed in Table 1. As shown in Table 1, all mice show close values of B(0.9758±0.03513) and $\beta (0.04558\pm 0.005207\;\min^{-1})$, suggesting that the pharmacokinetic parameters of ICG in liver exhibit a remarkable consistency within a specific kind of mice. The slight difference of the pharmacokinetic parameters may be caused by different experimental conditions and physiological status of the mice.

**Table 1:** List of ICG pharmacokinetic parameters in the liver of three mice.

No.	B (a.u.)	β ($\min^{-1}$)
1	0.9757	0.04249
2	0.9409	0.04347
3	1.0110	0.05079

## 4 Conclusions

We proposed a dynamic DFT system for achieving pharmacokinetic parameters of ICG in living mice. Under the consideration of the balance between cost and effectiveness, the system employs lock-in photon-counting detection of square wave modulation mode to provide high sensitivity, large dynamic-range, and high ability to reject ambient light. The comprehensive assessments of the system have been experimentally conducted on static phantoms, dynamic phantom, and *in vivo* mice. The static phantom with a single-target and three-target experiments validate the capability of the system to recover the locations and sizes of the targets, and demonstrate an excellent quantitativeness of the reconstructed fluorescence yield. Furthermore, the self-designed dynamic phantom experiments verified that the dynamic system can capture rapidly changing fluorescence signals. Finally, the *in vivo* experiments show the feasibility to acquire the time-course of ICG concentration images in health mice’s livers for pharmacokinetic analysis. These results demonstrate that our proposed system may provide a valuable tool for tumor detection, drug assessment, and liver function evaluation.

## Data Availability

Data underlying the results presented in this paper are not publicly available at this time but may be obtained from the authors upon reasonable request.
